# Urinary Lysosomal Enzyme Activities and Albuminuria in Ghanaian Patients with Type 2 Diabetes Mellitus

**DOI:** 10.1155/2016/2810639

**Published:** 2016-08-09

**Authors:** Henry Asare-Anane, Felix Twum, Emmanuel Kwaku Ofori, Erving L. Torgbor, Seth D. Amanquah, Charlotte Osafo

**Affiliations:** ^1^Department of Chemical Pathology, S.B.A.H.S, University of Ghana, Accra 00233, Ghana; ^2^Department of Physiology, University of Lausanne, 1015 Lausanne, Switzerland; ^3^Renal Unit, Korle-Bu Teaching Hospital, Accra, Ghana

## Abstract

Renal tubular lysosomal enzyme activities like alanine aminopeptidase (AAP) and N-acetyl-*β*-D-glucosaminidase (NAG) have been shown to increase in patients developing diabetic nephropathy and nephrosclerosis. This study aimed to determine the activities of N-acetyl-*β*-D-glucosaminidase and alanine aminopeptidase and albumin concentration in urine samples of patients with type 2 diabetes. One hundred and thirty (65 type 2 diabetic and 65 nondiabetic) subjects participated in this study. Blood samples were drawn for measurements of fasting blood glucose, albumin (Alb), lipids, and creatinine (Cr). Early morning spot urine samples were also collected for activities of alanine aminopeptidase (AAP), N-acetyl-*β*-D-glucosaminidase (NAG), and concentration of albumin (U-Alb) and creatinine (U-Cr). Both NAG/Cr and AAP/Cr were significantly increased in diabetic subjects compared to controls (*p* < 0.001). There was positive correlation between NAG/Cr and Alb/Cr (*r* = 0.49, *p* < 0.001) and between NAG/Cr and serum creatinine (*r* = 0.441, *p* < 0.001). A negative correlation was found between NAG/Cr and eGFR (*r* = −0.432, *p* < 0.05). 9.3% and 12% of diabetics with normoalbuminuria had elevated levels of AAP/Cr and NAG/Cr, respectively. We conclude that measuring the urinary enzymes activities (NAG/Cr and AAP/Cr) could be useful as a biomarker of early renal involvement in diabetic complications.

## 1. Introduction

Diabetic nephropathy (DN) and nephrosclerosis are clinical conditions characterized by persistent albuminuria, arterial blood pressure elevation, and an increasing decline in glomerular filtration rate (GFR) [1–4]. Patients with the above conditions represent major complications developed in diabetes mellitus and have a higher risk of developing cardiovascular diseases (CVD) [5–7]. This imposes enormous medical, economic, and social burden in terms of costs on both the patient and the health care system. Early clinical manifestation of diabetic nephropathy is the appearance of microalbuminuria, defined by a urinary albumin excretion rate of 30–300 mg/24 hrs or 30–300 mg/g of creatinine in a spot urine sample, which predicts renal function deterioration and poor outcome. Even though microalbuminuria is the most widely used test of development of diabetic nephropathy, it does not manifest clinically until stage 3 of the five stages of development of diabetic nephropathy [8]. During the development of DN, certain lysosomal enzymes are secreted into the urine. Lysosomal enzymatic activities have been shown to be elevated in urine samples of patients developing diabetic nephropathy earlier than appearance of microalbuminuria [9]. N-acetyl-*β*-D-glucosaminidase and alanine aminopeptidase are among the renal tubular lysosomal enzymes that have been shown to be excreted in higher concentrations during the early development of diabetic nephropathy. Early detection of renal involvement in DN, through urinary activities of alanine aminopeptidase (AAP) and N-acetyl-*β*-D-glucosaminidase (NAG), can draw the attention of clinicians to possible development of DN and therefore put in place appropriate therapeutic measures in order to prevent the disease progressing to end stage, thereby leading to increased survival and lower treatment cost. The objective of this study was to determine the activities of N-acetyl-*β*-D-glucosaminidase and alanine aminopeptidase and albumin concentration in urine samples of patients with type 2 diabetes mellitus.

## 2. Materials and Methods

### 2.1. Study Site, Design, Participants, and Exclusion Criteria

This was a hospital based cross-sectional study with matched controls, carried out at the National Diabetes Management and Research Centre (NDMRC), Korle-bu Teaching Hospital, Accra, Ghana, from October 2013 to July 2014. Subjects who served as controls were screened to make sure that they were nondiabetic. A total of 301 subjects were spoken to and 223 agreed to participate in the study. Detailed medical history and examination were conducted by a diabetologist to exclude subjects with conditions that could affect results outcome such as subjects with renal and liver diseases, proteinuria, pregnancy, immunosuppression, and urinary tract infections (UTI). Finally, 130 subjects comprising 65 type 2 diabetics and 65 healthy controls between the ages of 25 and 83 years were involved in the study. The University of Ghana Medical School Ethical and Protocol review committee reviewed and approved the study. Informed written consent was obtained from all participants in accordance with the Helsinki Declaration [10]. A questionnaire covering participants' lifestyle, dietary habits, anthropometrics, and medications used was administered to each participant that consented. Anthropometric measurements such as height and weight were taken and body mass index was calculated. Blood pressure was taken using a mercury sphygmomanometer and stethoscope after participants had rested for 15 minutes.

#### 2.1.1. Minimum Sample Size

Assuming odds ratio of 2 among diabetic subjects for albuminuria, at a 5% significant level and a power of 80%, a sample size of 60 persons was adequate for this study.

#### 2.1.2. Laboratory Procedure

This study used a morning spot urine sample as recommended by Kidney Disease Outcomes Quality Initiative (KDOQI). Early morning spot urine samples from study subjects were collected into sterile plastic universal urine containers. Samples were centrifuged at 2000 g for 10 minutes. Dipstick evaluation for proteinuria and urine analysis was performed on each sample. Five milliliters (5 mL) of venous blood sample was collected between 6 am and 8 am, after participants had fasted for 10–14 hours. Four milliliters (4 mL) of the blood sample was transferred into serum separator tubes and processed. The resulting sera after processing were aliquoted into Eppendorf tubes and stored at −20°C until required for use. The remaining 1 mL was transferred into sodium fluoride containing tube for the estimation of glucose. Fasting blood glucose (FBG), total cholesterol (TC), triglycerides (TG), high density lipoprotein cholesterol (HDL) creatinine (CR), albumin (Alb), and alanine aminotransferase (ALT) activity were analyzed using an Erba Mannheim Chem-7 spectrophotometer (India).

### 2.2. Statistical Analysis

Data was entered unto a spread sheet and analyzed using Microsoft Office Excel, 2013 (Microsoft Cooperation, Louiseville, USA), and the values were expressed as mean plus or minus standard deviations. The statistical Package for Social Sciences (SPSS) version 20.0 (SPSS Software, San Diego, USA) was used for most of the statistical and analytical work with a level of statistical significance set at *p* < 0.05 for all tests. The unpaired Student's* t*-test was used to evaluate differences between two means. Pearson product moment correlation coefficient (*r*) was used to find the association between two continuous variables.

## 3. Results

A total of 130 subjects took part in the study with age range of 25–83 years. The subjects were made up of 65 cases (diabetics) comprising 28 males (43%) and 37 females (57%) and 65 apparently healthy controls, consisting of 29 males (44.6%) and 36 females (55.4%). The clinical and biochemical parameters of the study population are shown in Table 1.

Diabetic subjects had significantly higher urine creatinine (U-Cr), N-acetyl-*β*-D-glucosaminidase (NAG), alanine aminopeptidase (AAP), urinary albumin to urinary creatinine ratio (U-Alb/U-Cr), NAG/U-Cr, and AAP/U-Cr compared to controls (*p* < 0.05). Urinary biochemical analytes for normoalbuminuric and microalbuminuric diabetic subjects are shown in Table 2. 9.3% and 12% of diabetics with normoalbuminuria had elevated levels of AAP/Cr and NAG/Cr, respectively.

Associations between correlates (eGFR, S-Cr, and duration of DM) with urinary analytes (Alb-Cr, NAG/Cr, and AAP/Cr) were determined among type 2 diabetic subjects and shown in Table 3. There were significant positive correlations for alb/Cr, NAG/Cr, and AAP/Cr with serum creatinine and a negative correlation between alb/Cr, NAG/Cr, and AAP/Cr and eGFR (Table 3).

## 4. Discussion

Diabetic nephropathy, which is one of the microvascular complications of diabetes mellitus, is the leading cause of end-stage renal disease in both the developed and developing countries [11]. Microalbuminuria is the most widely used clinical test for earliest renal involvement in diabetic nephropathy [5]. A number of studies, however, have shown that renal tubular lysosomal enzymes are elevated in urine before the onset of microalbuminuria [12, 13]. This study evaluated the urinary activities of two lysosomal enzymes (NAG and AAP) that are located along the brush border of the proximal tubules of the kidney.

In this study, the mean BMI of the diabetic subjects was significantly higher than that of their nondiabetic counterparts (Table 1). This result was in agreement with a study carried out in Kumasi, Ghana, by Danquah and colleagues [14]. This could be as a result of the association of diabetes mellitus with increased weight and obesity [14, 15]. There was no significant difference in both systolic and diastolic blood pressures for diabetic subjects and controls. This finding agreed with studies done elsewhere [13, 16] and could be a result of the fact that diabetic subjects were being managed on antihypertensive medications. Subjects in the control group were not on any form of medication. In addition, subjects within the control group who had elevated blood pressure did not have corresponding elevated urinary markers. This study also revealed a significantly higher concentration of total cholesterol (T.CHOL), LDL-cholesterol, and triglyceride in the diabetic group compared to controls.

HDL-cholesterol was, however, significantly higher in controls than diabetics. This lipid pattern agrees with earlier studies [17]. This is likely the result of the association of diabetes mellitus with increased dyslipidemia. Serum creatinine and estimated glomerular filtration rate (eGFR) were significantly higher and lower, respectively, in the type 2 diabetic subjects than their control counterparts. These results were in agreement with previous studies [9, 14, 16, 18].

The prevalence of microalbuminuria among the diabetics in this study was 40% and was similar to previous studies [14, 19]. Other studies in Tanzania [20] and Nigeria [21] had 29% and 52% prevalence, respectively. This variation could be attributed to factors such as sample size and ethnicity. Results from this study showed a significant increase in both urinary NAG activity and NAG/Cr in diabetics compared with controls. These corroborate work done elsewhere [13, 22, 23]. This study also found significant elevations in both urinary AAP activity and AAP/Cr in the diabetics compared with controls. The results agreed with studies done elsewhere [24–27]. Tubulointerstitial injuries of the kidney due to development of diabetic nephropathy in the diabetics might have caused the elevated NAG and NAG/Cr levels. Also, there were significant increases in NAG/Cr, AAP/Cr, and Alb/Cr in the diabetics with microalbuminuria compared with diabetics with normoalbuminuria (Table 2). This was in agreement with work done by Nikolov and friends [9]. This could be the result of increased tubular and glomerular lesions that led to leakage of the enzymes into urine due to persistent hyperglycemia in the microalbuminuric diabetics compared to the normoalbuminuric diabetics. This study also showed an association between correlates (eGFR, S-Cr, and duration of DM) and urinary parameters NAG/Cr, AAP/Cr, and Alb/Cr (Table 3). Findings are consistent with earlier research [22]. NAG/Cr would be a preferred marker for diagnosing diabetic nephropathy compared with AAP/Cr because it was much more elevated in diabetics compared with controls and also in terms of percentages among normoalbuminuric diabetics, NAG/Cr was higher than AAP/Cr. The presence of these elevated enzymes in normoalbuminuric patients is an indication of tubular destruction before the onset of microalbuminuria. Logistic and time constraints restricted this study to the selection of type 2 diabetic subjects only. In addition to the inclusion of type 1 diabetic subjects, we recommend a longitudinal study to investigate the clinical utility of NAG and AAP as a screening tool for early detection of diabetic complications.

## 5. Conclusion

Results from this study showed significantly elevated levels of urinary NAG, AAP, and albumin-creatinine ratio in the diabetic subjects as compared to their nondiabetic counterparts. Furthermore, there was a strong positive correlation of urinary enzyme activities with urinary albumin-creatinine ratio. Urinary activities of NAG and AAP together with their creatinine ratios could be used as markers of diabetic nephropathy.

## Figures and Tables

**Table 1:** Clinical and biochemical parameters of the study population.

Parameters	Diabetics (*N* = 65)	Controls (*N* = 65)	*p* value
Age (yrs)	55.3 ± 11.7	51.2 ± 11.5	0.061
SBP (mmHg)	147.8 ± 28.3	145.4 ± 21.8	0.388
DBP (mmHg)	88.8 ± 15.2	84.6 ± 10.6	0.079
BMI (kg/m^2^)	28.6 ± 4.7	26.2 ± 2.6	0.001
WHR (ratio)	0.88 ± 0.17	0.81 ± 0.11	0.230
FBG (mmol/L)	9.68 ± 3.4	5.8 ± 1.2	0.001
T.CHOL (mmol/L)	5.87 ± 1.1	4.88 ± 1.0	0.018
TG (mmol/L)	1.99 ± 0.7	1.60 ± 0.6	0.006
HDL (mmol/L)	1.58 ± 0.6	1.89 ± 0.7	0.006
LDL (mmol/L)	3.32 ± 0.9	2.63 ± 1.3	0.004
S-creatinine (*µ*mol/L)	98.8 ± 15.0	85.4 ± 10.9	0.001
eGFR (mL/min)	77.8 ± 10.8	93.6 ± 11.3	0.001
Period of diabetes (yrs)	8.2 ± 6.1	—	—

Urinary biochemical analytes of the study population			
U-creatinine (g/L)	2.1 ± 1.5	1.1 ± 0.8	0.001
U-albumin (mg/L)	46.1 ± 13.4	35.1 ± 12.8	0.092
NAG (U/L)	13.0 ± 2.2	10.1 ± 1.3	0.001
AAP (mg/L)	4.35 ± 1.3	3.89 ± 0.9	0.024
U-Alb/U-Cr (mg/g)	54.1 ± 14.9	17.8 ± 12.6	0.001
NAG/U-Cr (U/g)	17.9 ± 8.0	9.8 ± 5.4	0.001
AAP/U-Cr (ng/g)	5.74 ± 3.6	3.0 ± 2.4	0.001

Values are given as mean ± standard deviation. *p* < 0.05: mean difference is significant. *p* < 0.001: mean difference is highly significant. SBP is systolic blood pressure, DBP is diastolic blood pressure, WHR is waist hip ratio, T.CHOL is total cholesterol, TG is triglyceride, HDL is high density lipoprotein, LDL is low density lipoprotein, FBG is fasting blood glucose, BMI is body mass index, eGFR is estimated glomerular filtration rate, NAG is N-acetyl-*β*-D-glucosaminidase, and AAP is alanine aminopeptidase. U-Cr is urinary creatinine and U-Alb is urinary albumin.

**Table 2:** Urinary biochemical analytes of microalbuminuric and normoalbuminuric diabetics.

Variable	Normoalbuminuria (<30 mg/g)	Microalbuminuria (30–300 mg/g)	*p* value
U-creatinine (g/L)	0.76 ± 0.67	1.34 ± 0.78	0.001
U-albumin (mg/L)	22.4 ± 12.1	81.56 ± 70.3	0.001
NAG (U/L)	12.88 ± 2.07	13.28 ± 2.4	0.495
AAP (ng/mL)	4.34 ± 1.26	4.35 ± 1.32	0.989
Alb/Cr (mg/g)	17.67 ± 4.86	108.7 ± 50.1	0.001
NAG/Cr (U/g)	11.83 ± 6.76	26.7 ± 17.2	0.001
AAP/Cr (ng/g)	3.84 ± 1.91	8.6 ± 5.79	0.001

% normoalbuminuric diabetics with AAP/U-Cr and NAG/U-Cr			
AAP/U-Cr		9.3	
NAG/U-Cr		12.0	

Data is presented as mean ± SD and percentages. *p* values ≤ 0.05 were considered as statistically significant. NAG is N-acetyl-*β*-D-glucosaminidase, AAP is alanine aminopeptidase, Cr is creatinine, Alb is albumin, and U-Cr is urinary creatinine.

**Table 3:** Association of urinary analytes with creatinine, eGFR, and duration of diabetes.

Variable	eGFR	S-creatinine	Duration of DM	Alb/Cr
Alb/Cr (mg/g)	*r* = −0.302 *p* = 0.014	*r* = 0.25 *p* = 0.04	*r* = 0.062 *p* = 0.604	— —

NAG/Cr (U/g)	*r* = −0.432 *p* = 0.001	*r* = 0.441 *p* = 0.001	*r* = 0.015 *p* = 0.907	*r* = 0.49 *p* = 0.001

AAP/Cr (ng/g)	*r* = −0.352 *p* = 0.004	*r* = 0.303 *p* = 0.014	*r* = 0.065 *p* = 0.606	*r* = 0.43 *p* = 0.001

*p* values < 0.05 were considered statistically significant. Correlation coefficient *r* > 0.5 shows strong positive correlation, and *r* < 0.5 shows weak positive correlation. NAG is N-acetyl-*β*-D-glucosaminidase, AAP is alanine aminopeptidase, Cr is creatinine, Alb is albumin, and eGFR is estimated glomerular filtration rate.

## References

[1] American Diabetes Association (2008). Standards of medical care in diabetes—2008. *Diabetes Care*.

[2] Jawa A., Kcomt J., Fonseca V. A. (2004). Diabetic nephropathy and retinopathy. *Medical Clinics of North America*.

[3] Hovind P., Rossing P., Tarnow L., Smidt U. M., Parving H.-H. (2001). Progression of diabetic nephropathy. *Kidney International*.

[4] Bloomgarden Z. T. (2005). Diabetic nephropathy: American Diabetes Association statements. *Diabetes Care*.

[5] Gross J. L., De Azevedo M. J., Silveiro S. P., Canani L. H., Caramori M. L., Zelmanovitz T. (2005). Diabetic nephropathy: diagnosis, prevention, and treatment. *Diabetes Care*.

[6] Lehmann R., Spinas G. A., Schweiz R. (1995). Diabetic nephropathy: significance of microalbuminuria and proteinuria in Type 1 and Type 2 diabetes mellitus. *Medical Prax*.

[7] Adeghate E., Schattner P., Dunn E. (2006). An update on the etiology and epidemiology of diabetes mellitus. *Annals of the New York Academy of Sciences*.

[8] Mogensen C. E., Christensen C. K., Vittinghus E. (1983). The stages in diabetic renal disease, with emphasis on the stage of incipient diabetic nephropathy. *Diabetes*.

[9] Nikolov G., Boncheva M., Gruev T., Biljali S., Stojceva-Taneva O., Masim-Spasovska E. (2014). Urinary biomarkers in the early diagnosis of renal damage in diabetes mellitus patients. *Scripta Scientifica Medica*.

[10] Helsinki Protocol Declaration Ethical Principles for Medical Research. https://en.wikipedia.org/wiki/Declaration_of_Helsinki.

[11] Hess B. (1999). Pharmacologic treatment of hypertension in diabetic patients. *Therapeutische Umschau*.

[12] Gatsing D., Garba I. H., Adoga G. I. (2006). The use of lysosomal enzymuria in the early detection and monitoring of the progression of diabetic nephropathy. *Indian Journal of Clinical Biochemistry*.

[13] Udomah F. P., Udeme E. E., Efa E., Babatunde S., Ayodeji A., Kadiri S. (2012). Association between urinary N-acetyl-*β*-D-glucosaminidase and microalbuminuria in diabetic black Africans. *International Journal of Nephrology*.

[14] Danquah I., Bedu-Addo G., Terpe K.-J. (2012). Diabetes mellitus type 2 in urban Ghana: characteristics and associated factors. *BMC Public Health*.

[15] Hu F. B., Li T. Y., Colditz G. A., Willett W. C., Manson J. E. (2003). Television watching and other sedentary behaviors in relation to risk of obesity and type 2 diabetes mellitus in women. *The Journal of the American Medical Association*.

[16] Habib S. S., Aslam M., Hameed W. (2005). Gender differences in lipids and lipoprotein profiles in healthy individuals and patients with type 2 diabetes mellitus. *Pakistan Journal of Physiology*.

[17] Samatha P., Venkateswarlu M., Siva P. V. (2012). Lipid profile levels in type 2 diabetes mellitus from the tribal population of Adilabad in Andhra Pradesh, India. *Journal of Clinical and Diagnostic Research*.

[18] Park W. D., Larson T. S., Griffin M. D., Stegall M. D. (2012). Identification and characterization of kidney transplants with good glomerular filtration rate at 1 year but subsequent progressive loss of renal function. *Transplantation*.

[19] Eghan B. A., Frempong M. T., Adjei-Poku M. (2007). Prevalence and predictors of microalbuminuria in patients with diabetes mellitus: a cross-sectional observational study in Kumasi, Ghana. *Ethnicity and Disease*.

[20] Ghosh S., Lyaruu I., Yeates K. (2012). Prevalence and factors associated with microalbuminuria in type 2 diabetic patients at a diabetes clinic in northern Tanzania. *African Journal of Diabetes Medicine*.

[21] Erasmus R. T., Oyeyinka G., Arijel A. (1992). Microalbuminuria in non-insulin-dependent (type 2) Nigerian diabetics: relation to glycaemic control, blood pressure and retinopathy. *Postgraduate Medical Journal*.

[22] Nakamura S. (1991). Clinical evaluation of urinary alanine aminopeptidase in the patients with diabetes mellitus: comparison among AAP microalbumin and N-acetyl-beta-D-glucosaminidase. *Hokkaido Igaku Zasshi*.

[23] Ambade V., Singh P., Somani B. L., Basannar D. (2006). Urinary N-acetyl beta glucosaminidase and gamma glutamyl transferase as early markers of diabetic nephropathy. *Indian Journal of Clinical Biochemistry*.

[24] Vlaskou D., Hofmann W., Guder W. G., Siskos P. A., Dionyssiou-Asteriou A. (2000). Human neutral brush border endopeptidase EC 3.4.24.11 in urine, its isolation, characterisation and activity in renal diseases. *Clinica Chimica Acta*.

[25] Stammberger K., Pfaffenberg R., Thulin H. (1975). Urinary excretion of analine aminopeptidase and lysozyme in diabetes. *Zeitschrift fur die Gesamte Innere Medizin und Ihre Grenzgebiete*.

[26] Shimojo N., Kitahashi S., Naka K. (1987). Comparison of N-acetyl-*β*-D-glucosaminidase and alanine aminopeptidase activities for evaluation of microangiopathy in diabetes mellitus. *Metabolism*.

[27] Srikrishna K., Kanagasabapathy A. S., John L. (1994). N-acetyl-*β*-D-glucosaminidase, alanine aminopeptidase and protein: creatinine ratio as early indicators of diabetic microangiopathy. *Indian Journal of Clinical Biochemistry*.

